# Broadband angle- and permittivity-insensitive nondispersive optical activity based on planar chiral metamaterials

**DOI:** 10.1038/s41598-017-11242-9

**Published:** 2017-09-06

**Authors:** Kun Song, Zhaoxian Su, Min Wang, Sinhara Silva, Khagendra Bhattarai, Changlin Ding, Yahong Liu, Chunrong Luo, Xiaopeng Zhao, Jiangfeng Zhou

**Affiliations:** 1 Department of Applied Physics, Northwestern Polytechnical University, Xi’an, 710129 China; 2 Department of Physics, University of South Florida, 4202 East Fowler Ave, Tampa, FL 33620-5700 USA

## Abstract

Because of the strong inherent resonances, the giant optical activity obtained via chiral metamaterials generally suffers from high dispersion, which has been a big stumbling block to broadband applications. In this paper, we propose a type of planar chiral metamaterial consisting of interconnected metal helix slat structures with four-fold symmetry, which exhibits nonresonant Drude-like response and can therefore avoid the highly dispersive optical activity resulting from resonances. It shows that the well-designed chiral metamaterial can achieve nondispersive and pure optical activity with high transmittance in a broadband frequency range. And the optical activity of multi-layer chiral metamaterials is proportional to the layer numbers of single-layer chiral metamaterial. Most remarkably, the broadband behaviors of nondispersive optical activity and high transmission are insensitive to the incident angles of electromagnetic waves and permittivity of dielectric substrate, thereby enabling more flexibility in polarization manipulation.

## Introduction

Manipulating the polarization states of electromagnetic waves has been a long-time interest in realms of life sciences, photoelectrons, telecommunications, *etc*. Materials with chirality, which can rotate the polarization plane of electromagnetic waves, are very suitable for designing polarization converters. However, the chirality in natural materials is usually extremely weak and dispersive, the polarization converters made of natural materials are unrealistic because of huge thicknesses being much larger than the operating wavelengths or narrow operating bandwidths, especially at gigahertz, terahertz and optical frequencies. Thus, materials that possess strong chirality are highly desired.

Metamaterials enable numerous of extraordinary electromagnetic phenomena that do not exist in natural materials, for instance, abnormal refraction or reflection^1–6^, super-resolution imaging^7, 8^, cloaking^9–11^, and perfect absorption of electromagnetic waves^12–14^. The presence of metamaterials makes it possible for us to obtain strong chirality. In the past few years, chiral metamaterials (CMMs) have been constructed to realize negative refractive index^15–20^, strong optical activity^21–25^, circular dichroism^26–30^, as well as asymmetric transmission^31–35^. Using strong resonances, the optical rotatory power of CMMs might rise to several orders of magnitudes larger than that of natural materials^36–40^. However, owing to the inherent Lorentz-like resonances, the giant optical activity of the previous CMMs is generally accompanied by high losses, high dispersion, narrow transmission bandwidths, and strong polarization distortion because of large ellipticity^18–20, 36, 41–43^, which is exceedingly detrimental for designing broadband and efficient polarization rotators. Recently, there has been much effort devoted to exploring nondispersive optical activity. By combining a meta-atom with its complement in a chiral configuration, low dispersive optical activity at the transmission resonance has been demonstrated; while this type of CMMs are subjected to narrow transmission bandwidths^44–46^. In more recent papers, three-dimensional off-resonant or nonresonant types of CMMs were also demonstrated to achieve nondispersive optical activity^47, 48^. However, the complicated fabrication process needed for creating these three-dimensional structures are very challenging even with current state-of-the-art technologies, especially at the optical part of the spectrum. Therefore, CMMs with simplified architectures that can be easily made using standard micro- or nano-fabrication processes are highly demanded.

In this paper, we propose a CMM working at GHz frequencies that is composed of a bi-layer planar helix structures with simple geometry where the interconnection between the bi-layer can be easily realized. Compared to previous work^48^, our design can be scale down to make CMMs operating in THz and even infrared regime utilizing standard micro- or nanofabrication techniques. Unlike the Lorentz-like resonances in the previous resonator based CMMs^16–20, 28, 36–38, 41, 42^, the effective permittivity of present CMM exhibits nonresonant Drude-like response, i.e. like that of plasma media, due to the continuity of the metallic structures. Simulation, calculated, and experimental results show that our CMM exhibits strong nondispersive optical activity, high transmittance and extremely low ellipticity in a broadband frequency range. And the optical activity of multilayer CMMs is proportional to the number of CMM layers. More interestingly, the nondispersive optical activity effect is independent of the incident angle and the dielectric constant of the substrate, which has not been reported previously to the best of our knowledge. Moreover, it is also found that the frequency of the transmission peak of our CMM presents dynamical tunability by altering the permittivity of dielectric substrate, indicating that the CMM is suitable for designing frequency-tunable polarization manipulation devices for telecommunication applications.

## Results

### Design of unit cells and theoretical calculation

**Figure 1:**
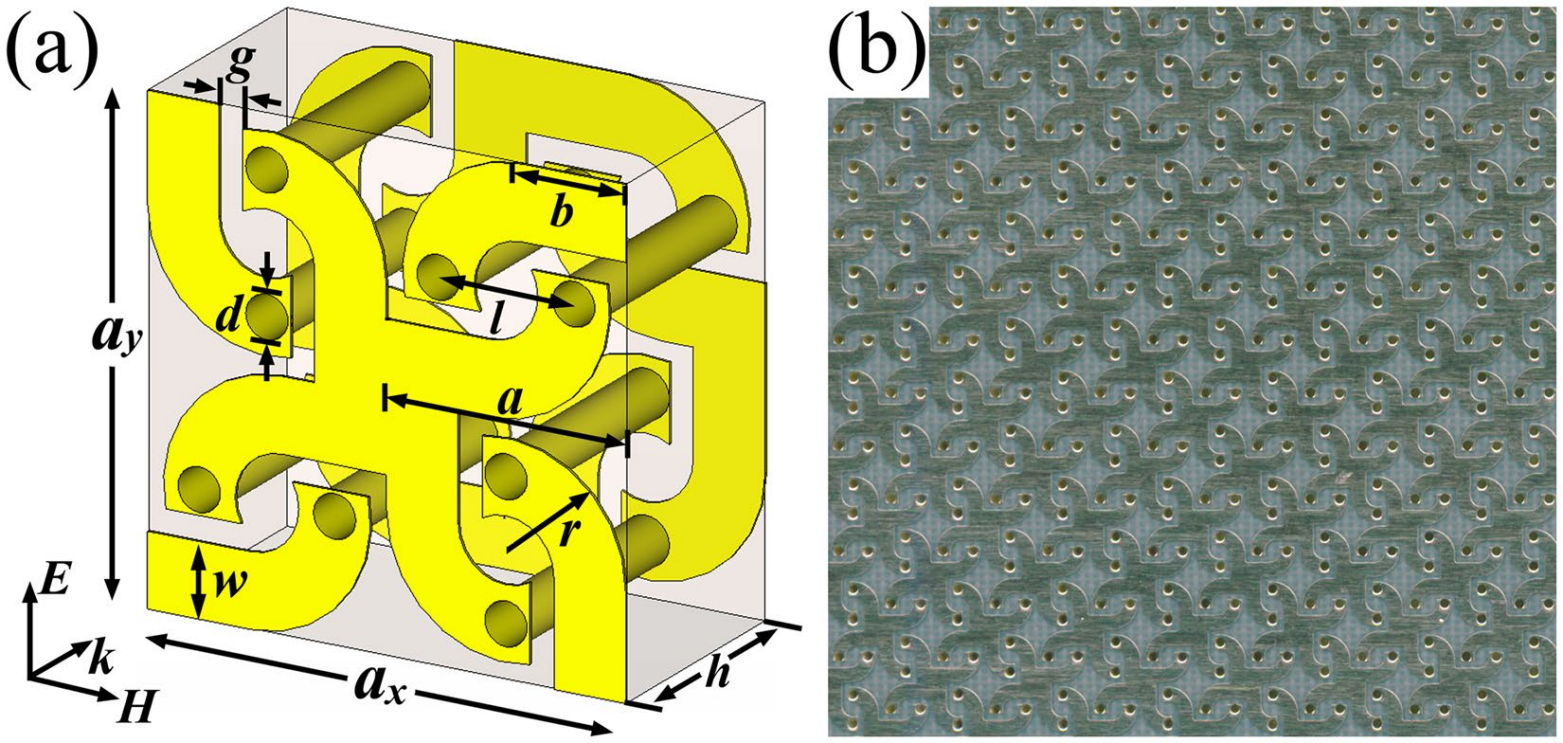
(**a**) Schematic diagram and (**b**) photograph of the proposed CMM.

Figure 1(a,b) show the schematic view and photograph of the present CMM, respectively. As shown in Fig. 1(a), the unit cell is composed of four interconnected double-helix structures. Along the clockwise direction, each double-helix are rotated by π/2 from its adjacent neighbor. Array of unit cells forms a C4 square lattice with four-fold symmetry which ensures a pure optical activity effect. The metal slices on the front and back surfaces of the dielectric substrate are connected via metallization holes. Obviously, all the unit cells of the designed CMM are interconnected. The geometrical parameters of the unit cells are as follows: *a*_*x*_ = *a*_*y*_ = 2*a* = 6 mm, *b* = 1.3 mm, *d* = 0.6 mm, *g* = 0.3 mm, *l* = 1.7 mm, *r* = 1.5 mm, *h* = 3.07 mm, and *w* = 0.9 mm. The metal copper cladding is 0.035 mm in thickness with a conductivity of *σ* = 5.8 × 10^7^ S/m. The permittivity of the F4BM-2 substrate is 2.65 + 0.001* *i*. In the experiments, a CMM sample with 40*40 unit cells was fabricated via the printed circuit board etching process.

To get the electromagnetic properties of the present CMM theoretically, we use the effective current model to calculate the effective parameters of the CMM^48^. Due to the interconnected metal structures, the currents can flow freely in the CMM, which will lead to a Drude-like response. For the double helix structure, the inductance and resistance are approximatively calculated as ${L}={{\mu }}_{0}{R}(\mathrm{ln}\,\frac{8{R}}{w}-2)$ and ${R}_{{\rm{\Omega }}}=\sqrt{\frac{{\mu }_{0}\omega S}{2\sigma {w}^{2}}}$, respectively, where $R{=}\sqrt{S}{=}\sqrt{(l+d)h}$, *S* is the effective cross section area of the double helix structure^48–50^. The electric potential of time-harmonic electromagnetic field can be expressed as:

1 $$\xi =aE-(\pm i{\omega }{{\mu }}_{0}{SH})=({{R}}_{{\rm{\Omega }}}-i{\omega }L){I}{.}$$

Here, the signs ± represent the right-handed and left-handed helix structures; *I* is the total current flowing in the helix structure. From Eq. 1, we can obtain:

2 $$I=\frac{aE}{{R}_{{\rm{\Omega }}}-i\omega L}-\frac{\pm i\omega {\mu }_{0}SH}{{R}_{{\rm{\Omega }}}-i\omega L}.$$

Then, the electric and magnetic dipole moments can be obtained via the following formulas^48^:

3 $${\boldsymbol{P}}=\frac{{\boldsymbol{J}}}{-i\omega }=\frac{{a}{\boldsymbol{I}}}{-i\omega V}=-\frac{{a}^{2}}{(i\alpha \omega +{\omega }^{2})LV}{\boldsymbol{E}}-\frac{\pm {\mu }_{0}aS}{(\alpha -i\omega )LV}{\boldsymbol{H}},$$

4 $${\boldsymbol{M}}=\pm \frac{{I}{\boldsymbol{S}}}{V}=\frac{\pm aS}{(\alpha -i\omega )LV}{\boldsymbol{E}}-\frac{i\omega {\mu }_{0}a{S}^{2}}{(\alpha -i\omega )LV}{\boldsymbol{H}},$$

where *V* is the volume of the unit cell and $\alpha =\frac{{R}_{{\rm{\Omega }}}}{L}$ is the dissipation constant. From Eqs 3 and 4, the electric displacement vector ***D*** and magnetic flux density vector ***B*** can be expressed as follows:

5 $${\boldsymbol{D}}={\varepsilon }_{0}{\boldsymbol{E}}+{\boldsymbol{P}}=[{\varepsilon }_{0}-\frac{{a}^{2}}{(i\alpha \omega +{\omega }^{2})LV}]{\boldsymbol{E}}-\frac{\pm {\mu }_{0}aS}{(\alpha -i\omega )LV}{\boldsymbol{H}},$$

6 $${\boldsymbol{B}}={\mu }_{0}({\boldsymbol{H}}+{\boldsymbol{M}})=\frac{\pm {\mu }_{0}aS}{(\alpha -i\omega )LV}{\boldsymbol{E}}+{\mu }_{0}{\boldsymbol{H}}-\frac{i\omega {\mu }_{0}^{2}a{S}^{2}}{(\alpha -i\omega )LV}{\boldsymbol{H}}.$$

For the chiral media, the constitutive equation is as follows:

7 $$(\begin{array}{c}{\boldsymbol{D}}\\ {\boldsymbol{B}}\end{array})=(\begin{array}{cc}{\varepsilon }_{0}\varepsilon  & -i{\kappa }{/}c\\ i{\kappa }{/}c & {\mu }_{0}\mu \end{array})(\begin{array}{c}{\boldsymbol{E}}\\ {\boldsymbol{H}}\end{array}),$$

where *κ* is the chirality parameter. Using Eqs 5–7, the effective parameters of the proposed CMM can be derived as follows:

8 $$\varepsilon ={\varepsilon }_{f}-\frac{{a}^{2}}{({\omega }^{2}+i\omega \alpha ){\varepsilon }_{0}LV},$$

9 $$\mu ={\mu }_{f}-\frac{\omega {\mu }_{0}{S}^{2}}{(\omega +i\alpha )LV},$$

10 $${\kappa }=\pm \frac{{\mu }_{0}caS}{(\omega +i\alpha )LV},$$

where ${\varepsilon }_{f}$ and ${\mu }_{f}$ are the quantitative fitting parameters. The transmission spectrum of the CMM is expressed in the terms of the following equation^48^:

11 $${T}=\frac{4Z{e}^{in{k}_{0}h}}{{(1+Z)}^{2}-{(1+Z)}^{2}{e}^{2in{k}_{0}h}}.$$

Here, $Z=\sqrt{\mu /\varepsilon }$ is the effective impedance; $n=\sqrt{\mu \varepsilon }$ is the effective average refractive index; ${k}_{0}=\omega \sqrt{{\mu }_{0}{\varepsilon }_{0}}$ is the wave number in vacuum. And the polarization azimuth rotation angle and ellipticity can be respectively calculated as:

12 $$\theta =\mathrm{Re}({\kappa })\cdot {k}_{0}h=\frac{\mathrm{Re}({\kappa })\omega h}{c}=\frac{{\mu }_{0}S}{4aL}\cdot \frac{{\omega }^{2}}{{\omega }^{2}-{\alpha }^{2}},$$

13 $$\eta =\text{Im}({\kappa })\cdot {k}_{0}h=\frac{\text{Im}({\kappa })\omega h}{c}=\frac{{\mu }_{0}S}{4aL}\cdot \frac{\omega \alpha }{{\omega }^{2}-{\alpha }^{2}}.$$

When the dissipation constant α ≪ ω, we can obtain:

14 $$\theta =\frac{{\mu }_{0}S}{4aL},$$

15 $$\eta =0.$$

### Results of single-layer CMM

**Figure 2:**
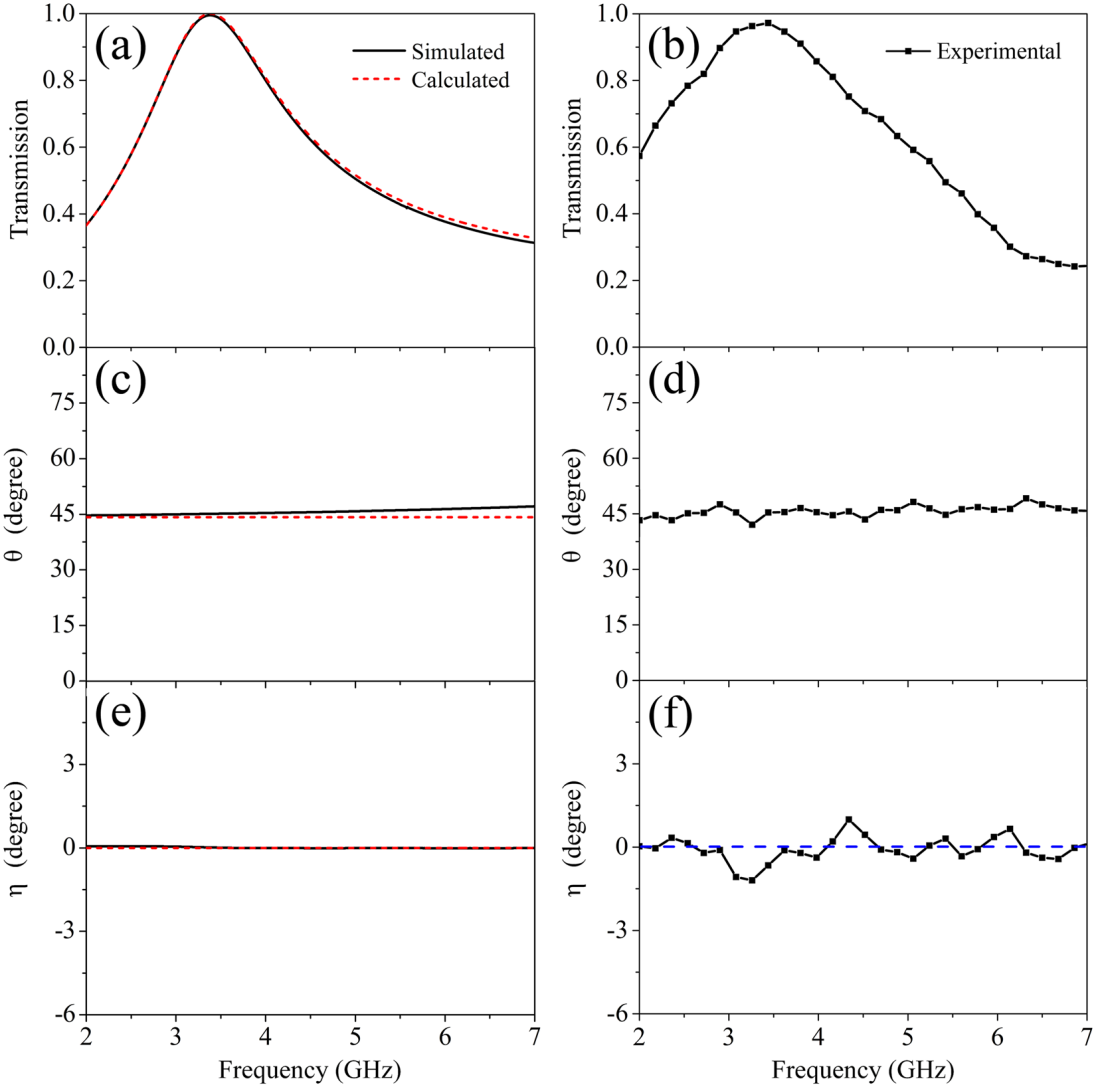
The simulated, calculated, and experimental results of the single-layer CMM at normal incidence. (**a**,**b**) Transmission spectra $(T=\sqrt{{T}_{xx}^{2}+{T}_{yx}^{2}})$, (**c**,**d**) Polarization azimuth rotation angle *θ*, (**e**,**f**) Ellipticity *η*. In the calculation, the quantitative fitting parameters ${\varepsilon }_{f}$ and ${\mu }_{f}$ are chosen as 21.3 and 0.95, respectively.

Figure 2 shows the simulated, calculated and measured results of the single-layer CMM at normal incidence. We can see clearly that the experimental results are in good qualitative agreement with the simulated and calculated ones. Figure 2(a,b) show that transmission reaches to the peak value of nearly unity at 3.4 GHz. Most importantly, the transmittance is over 0.8 in the wide frequency range of 2.9~4.0 GHz with a relative bandwidth of 32%, indicating that the present CMM can achieve broadband and high-efficiency transmission. The polarization azimuth rotation angle of the CMM are plotted in Fig. 2(c,d). Our sample shows a nearly constant rotation angle ~45° within the entire measured frequency region, indicating a broadband and nondispersive optical activity. The rotation angle can be controlled by using different geometric parameters, *l*, *a* and *h* as shown in Eq. 14. The dependences of *θ* on *a* and *h* are shown in supplementary material (see Fig. S1). Figure 2(e,f) depict that *η* is nearly zero over the entire frequency range. According to Eqs 13 and 15, η = 0 results from $\alpha \ll \omega$, indicating that our metamaterial exhibits very low loss. *η* = 0 also shows a purely optical activity effect^19^, i.e. the transmitted EM wave maintains its original polarization state without any distortion. As a comparison, large rotation angles, *θ*, obtained by resonator based CMMs^16–21, 28, 36–38, 41, 42^ are always accompanied by large *η*.

**Figure 3:**
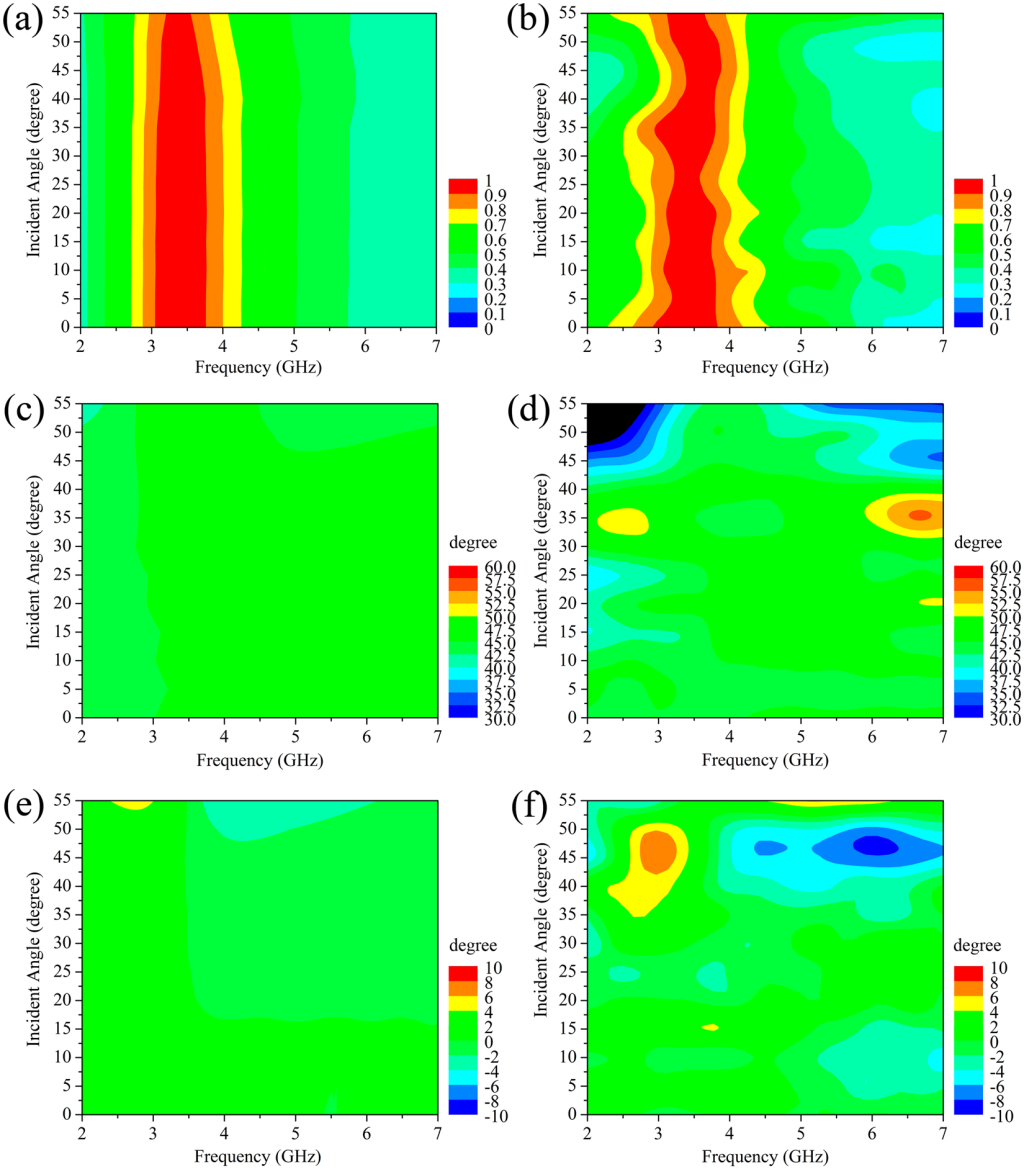
The simulation (left column) and experimental (right column) results of the single-layer CMM at oblique incidence. (**a**,**b**) Transmission spectra, (**c**,**d**) Polarization azimuth rotation angle *θ*, (**e**,**f**) Ellipticity *η*.

The electromagnetic properties of the single-layer CMM at oblique incidence are shown in Fig. 3. The incident angle of electromagnetic wave is tuned by the step of 5°. Figure 3(a,b) depict the simulation and experimental transmission spectra evolving with different incident angles. It shows that, although the transmission peak shows a very slight blue-shift and a slight reduction of the peak value as the incident angle increases, the broadband and high transmission features still exist. In Fig. 3(c,d), it is of significance that the polarization azimuth rotation angle *θ* in the whole frequency region is kept approximately constant at 45° with the incident angle increasing from 0° to 40°, which implies that the single-layer CMM can realize nondispersive optical activity in a wide range of incident angle. As shown in Fig. 3(e,f), the ellipticity of the single-layer CMM gradually increases with response to the increment of the incident angle. However, the maximum value of the ellipticity is less than 1.0° even if the incident angle rises up to 40°. As the ellipticity is very small, the transmission spectrum of the single-layer CMM can still be regarded as linearly polarized. These facts reveal that the broadband high transmission and nondispersive optical activity behaviors of the single-layer CMM can be maintained regardless of the incident angles, exhibiting more advantages than the CMMs previously reported^18–20, 36–38, 41, 42^.

**Figure 4:**
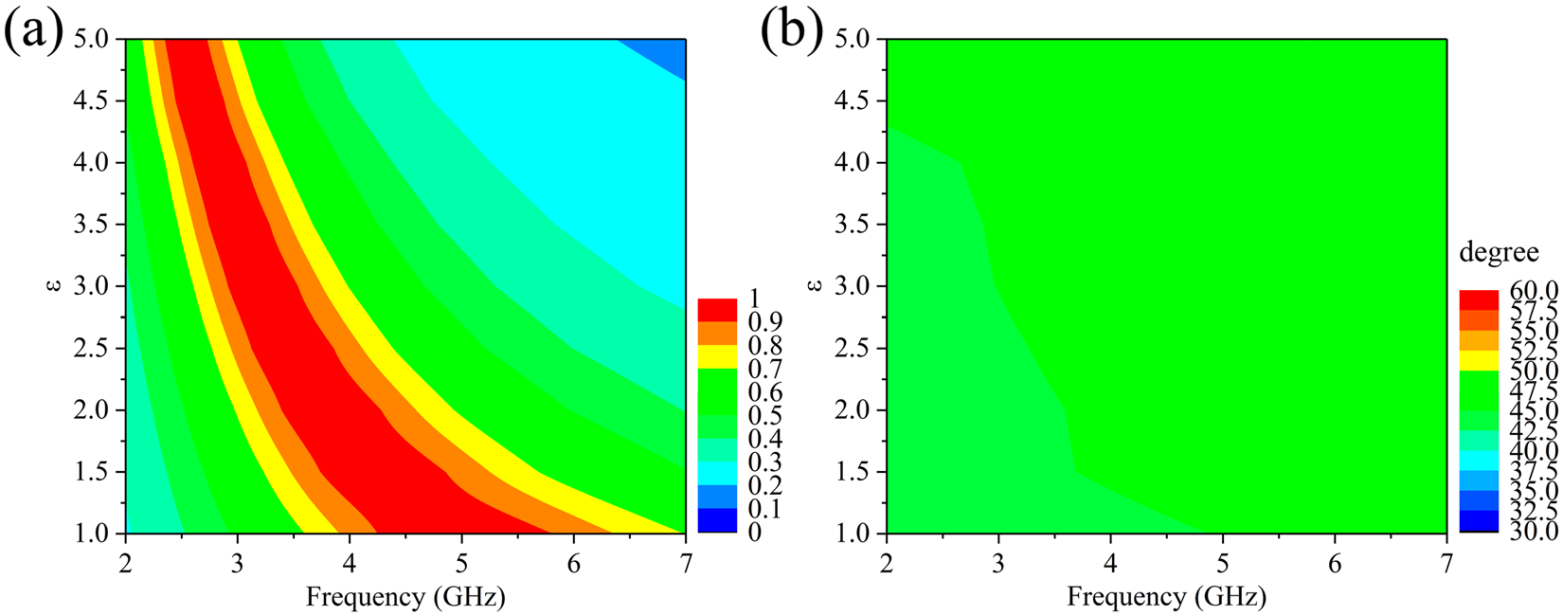
The simulated (**a**) transmission spectra and (**b**) polarization azimuth rotation angle of the single-layer CMM with different dielectric constants of the substrates. The dielectric constant of the substrate is tuned by a step of 0.5.

Generally, the electromagnetic manifestations of metamaterials can be profoundly affected by the permittivity of dielectric substrates. In Fig. 4, further simulations are carried out to investigate the influence of the permittivity of dielectric substrate on the transmission and optical activity of the single-layer CMM. Figure 4(a) portrays the transmission spectrum of the CMM evolving with different permittivity. The results show that, with the permittivity of dielectric substrate increasing, the transmission peak generates significant red shift and the relative transmission bandwidth with transmittance over 0.8 gradually decreases. It is noteworthy that, in spite of the decrement of relative transmission bandwidth, the CMM can still accomplish broadband and high-performance transmission when the permittivity increases. The red shift of transmission peak and the decrement of transmission bandwidth are attributed to that, the variation of permittivity of dielectric substrate leads to the changes of effective permittivity and permeability of the CMM, which further result in the alterations of the effective refractive index and impedance (see Figs S2–S5 in Supplementary). Consequently, the transmission spectrum will be changed significantly according to Eq. 11.

The effect of permittivity of substrate on the optical activity of the single-layer CMM is shown in Fig. 4(b). It is noteworthy that the polarization azimuth rotation angle of the CMM has been kept constant at about 45° over the all frequency range, without being influenced by the variation of permittivity. This phenomenon can be well explained by Eqs 12 and 14, from which we can see that the polarization azimuth rotation angle is unrelated to the permittivity of dielectric substrate. The aforementioned results confirm that the CMM can realize broadband nondispersive optical activity with high transmission, independent of permittivity of dielectric substrate. And these intriguing properties also imply that the CMM might be a good candidate for designing frequency-tunable polarization rotator.

### Results of dual-layer CMMs

**Figure 5:**
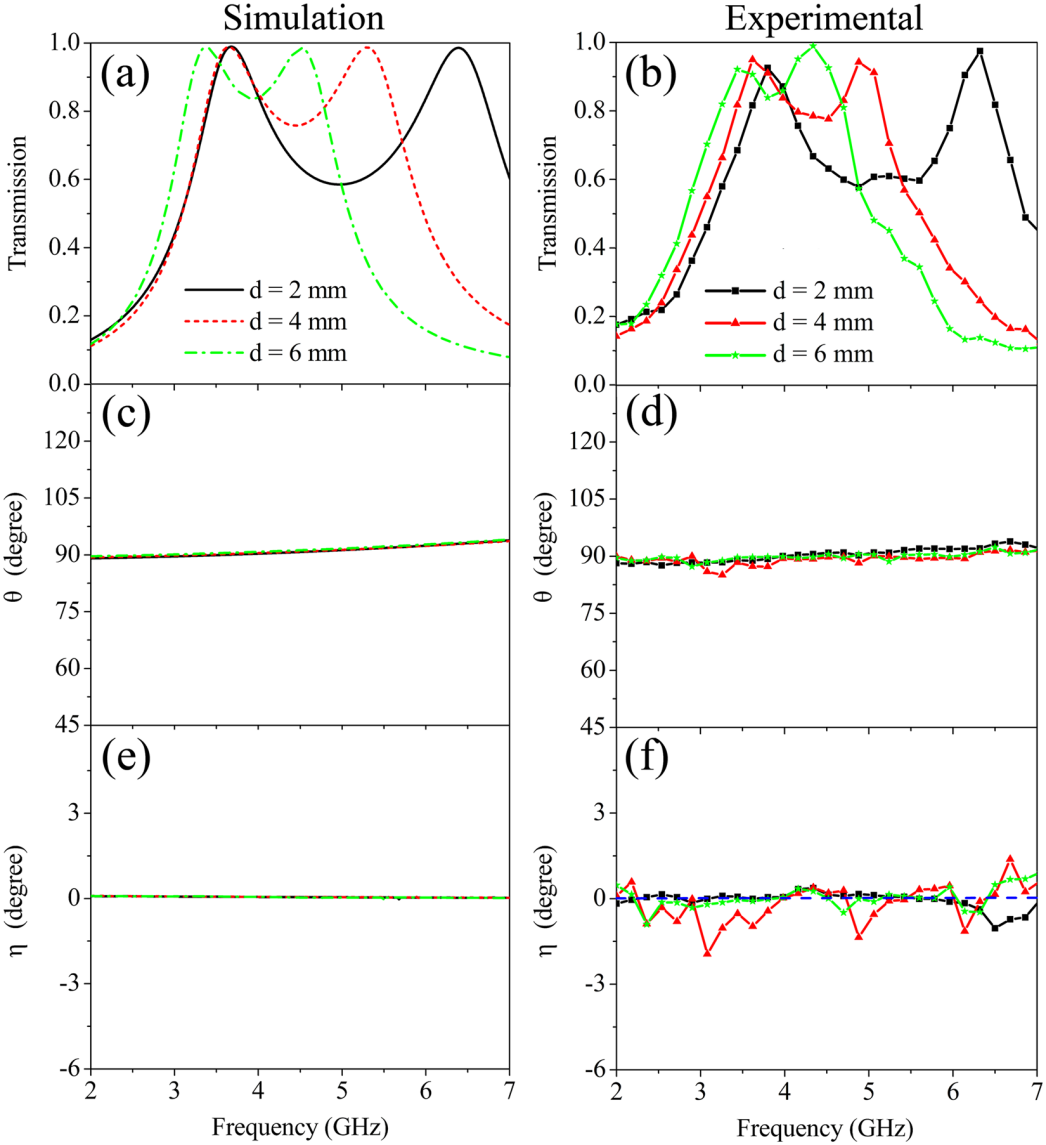
The numerical (left column) and measured (right column) results of the dual-layer CMMs with different interlayer spacings at normal incidence. (**a**,**b**) Transmission spectra, (**c**,**d**) Polarization azimuth rotation angle *θ*, (**e**,**f**) Ellipticity *η*.

The numerical and measured results of the dual-layer CMMs with different interlayer spacings at normal incidence are shown in Fig. 5. It is seen in Fig. 5(a,b) that there are two transmission peaks occurring on the transmission spectra for the dual-layer CMMs. As the interlayer spacing increases, the two transmission peaks exhibit significant red shifts and come close to each other. These phenomena arise due to the double layers of CMMs that form a Fabry-Pérot-like resonant cavity. The Fabry-Pérot-like resonance will occur when the electromagnetic waves are reflected between the double layers of CMMs, resulting in the generation of two transmission peaks^48^. Moreover, the Fabry-Pérot-like resonant cavity with different space distances will generate different resonant responses, which leads to the transmission spectra altering with the interlayer spacing^48^.

Figure 5(c–f) show the polarization azimuth rotation angle and ellipticity of dual-layer CMMs evolving with different interlayer spacings, respectively. It is obvious that the polarization azimuth rotation angle and ellipticity within the whole frequency region have been kept approximatively at 90° and 0°, respectively, and the alteration of the interlayer spacing has almost no influence on the optical activity and ellipticity of the dual-layer CMMs. The fascinating properties mentioned above imply that the dual-layer CMMs can function as a 90° polarization rotator, of which the transmission spectrum can be dynamically tuned by varying the space distance between the two layers of CMMs. Furthermore, compared Fig. 5(c,d) with Fig. 2(c,d), it can be found that the optical activity of dual-layer CMMs is just two times of that of the single-layer CMM. More simulations demonstrate that the optical activity of multi-layer CMMs is proportional to the number of CMM layers (see Fig. S6 in Supplementary).

## Discussion

**Figure 6:**
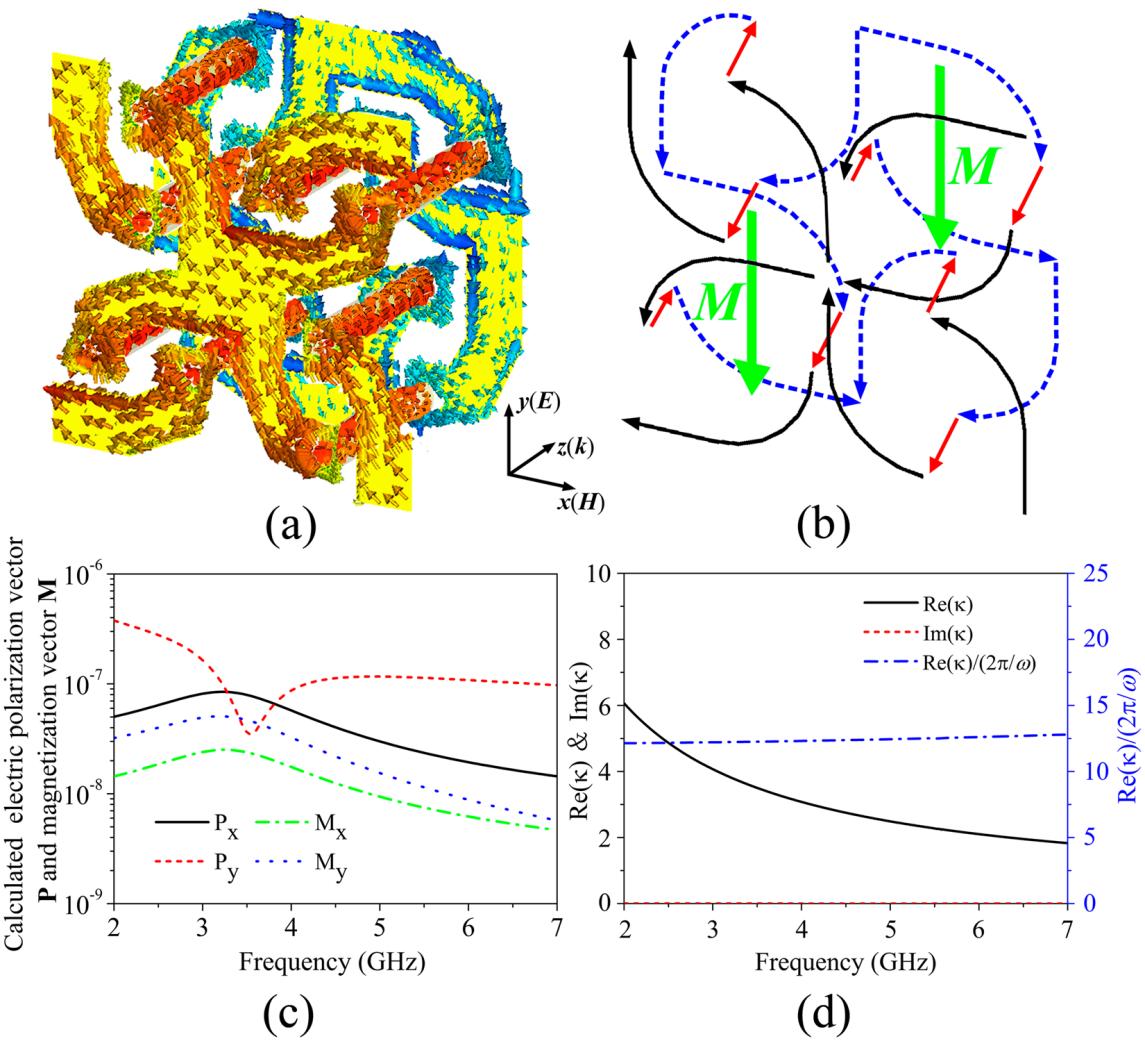
(**a**) Surface current distribution of the designed CMM at 3.4 GHz. (**b**) Schematic illustration of the flowing direction of surface currents. (**c**) The calculated electric polarization vector ***P*** and magnetization vector ***M***. Here, the *P*_*x*_ (*P*_*y*_) and *M*_*x*_ (*M*_*y*_) represent the *x*(*y*)-components of electric polarization vector and magnetization vector, respectively. (**d**) The numerically calculated results of Re(*κ*), Im(*κ*), and Re(*κ*) * *ω*/2π.

To get physical insight into the mechanism of broadband nondispersive optical activity and zero ellipticity, we examined the simulated surface current distributions of the considered CMM, as shown in Fig. 6(a). It is evident that the currents flow freely in the interconnected double-layer metal patterns, forming continuous currents in the 2D mesh network. Consequently, the CMM exhibits a nonresonant Drude-like response, which can effectively avoid the dispersive optical activity due to the Lorenz-like resonances in the previous CMMs^16, 19–21^. Figure 6(b) presents the schematic illustration of the flowing direction of surface currents. It is seen that the currents on the top (black solid lines) and bottom (blue dash lines) layers flow along the opposite directions. As a result, strong magnetic moments ***M*** are induced in the opposite direction to the external electric field ***E***. Therefore, the electric field ***E*** contributes to the magnetic field ***B*** as described in the constitutive equation (Eq. 7). This is the origin of the chirality of the proposed CMM. To further elucidate the chirality phenomenon, we calculated the electric polarization vector and magnetization vector, ***P*** and ***M***, respectively, using the volume current density distribution^51^. Figure 6(c) shows the *y*-components of ***P*** and ***M*** when the electric field, ***E***, of incident wave is linearly polarized along *y*-direction (and ***H*** is along *x*-direction). According to Eqs 5 and 6, we can find that *P*_*y*_ and *M*_*y*_ are induced by ***E*** field of the incident wave, while *P*_*x*_ and *M*_*x*_ are induced by ***H*** field. It is clearly shown that strong magnetic dipole moment *M*_*y*_ due to the electric field and strong electric dipole moment *P*_*x*_ due to the magnetic field are induced, and thereby confirming the origin of chirality. Figure 6(d) plots the numerical results of Re(*κ*), Im(*κ*), and Re(*κ*) * *ω*/2π. Our calculated result confirms that the dissipation constant *α* in Eq. 10 is far smaller than the angular frequency *ω*. As the chirality parameter *κ* is a finite value, the imaginary part of *κ* is thus nearly zero and can be ignored. Then, the Eq. 10 can be approximatively expressed as ${\kappa }\approx \pm \frac{{\mu }_{0}caS}{\omega LV}\approx \mathrm{Re}({\kappa })$, from which we can found that the real part of *κ* is inversely proportional to *ω*. As a result, the value of $\mathrm{Re}({\kappa })\ast \omega$ is a constant. Finally, the polarization azimuth rotation angle *θ* will be constant in the whole frequency range according to Eq. 14, *i*.*e*., the nondispersive optical activity occurs. Additionally, the circular dichroism of CMMs is closely related to the imaginary part of *κ*. Since imaginary part of *κ* is approximatively zero, the circular dichroism is therefore absent, which results in the ellipticity being zero.

## Conclusions

In summary, we have demonstrated a fascinating CMM that consists of planar helical structures. As a result of the interconnected metal structures, this kind of CMM generates Drude-like response, which combines with the C4 symmetry geometry giving rise to the broadband nondispersive optical activity and zero ellipticity simultaneously accompanied by high transmittivity. Most notably, the broadband behaviors of nondispersive optical activity and high transmission of the single-layer CMM are independent of the incident angles of electromagnetic waves and permittivity of dielectric substrate. And the transmission spectrum of the CMM can be successively tuned by varying the permittivity of substrate but with the optical activity unchanged. In addition, the optical activity of multi-layer CMMs is proportional to the number of CMM layers without being affected by the interlayer coupling effect, which enables us to obtain much stronger optical activity just by simply increasing the layer number. With the intriguing properties, the elaborate CMM exhibits more application flexibility and is greatly appealing for controlling the polarization states of electromagnetic waves.

## Methods

### Numerical simulation

Simulations were performed with the commercial software CST Microwave Studio. In the simulations, a linearly polarized wave was incident on the sample; the unit cell boundary conditions were employed in the *x* and *y* directions and open boundary conditions were utilized in the *z* direction.

### Experimental measurement

The experimental measurements were carried out by using an AV3629 network analyzer with two broadband linearly polarized horn antennas in an anechoic chamber. Using the linear co-polarization and cross-polarization transmission coefficients *T*_*xx*_ and *T*_*yx*_, we can obtain the transmission coefficients of the circularly polarized waves by the formula *T*_±_ = *T*_*xx*_ ± *i* * *T*_*yx*_, where *T*_+_ and *T*_−_ are the transmission coefficients of the right-handed and left-handed circularly polarized waves, respectively. The optical activity is revealed via the polarization azimuth rotation angle $\theta =\frac{\text{arg}({T}_{+})-\text{arg}({T}_{-})}{2}$. And the circular dichroism of the transmitted waves is characterized by the ellipticity $\eta =\frac{1}{2}\mathrm{arc}\,\tan \frac{{|{T}_{+}|}^{2}-{|{T}_{-}|}^{2}}{{|{T}_{+}|}^{2}+{|{T}_{-}|}^{2}}$.

## Electronic supplementary material

Supplementary Materials
