# Supplementary material for: Broadband angle- and permittivity-insensitive nondispersive optical activity based on planar chiral metamaterials
Source: Sci Rep. 2017 Sep 6;7:10730. doi: 10.1038/s41598-017-11242-9 (PMC5587580; doi:10.1038/s41598-017-11242-9)
Supplement: Supplementary file 1 — Supplementary Materials [file 41598_2017_11242_MOESM1_ESM.pdf]

Broadband angle- and permittivity-insensitive nondispersive optical activity based on planar chiral metamaterials

Kun Song,[1] Zhaoxian Su,[1] Min Wang,[1] Sinhara Silva[2], Khagendra Bhattarai[2], Changlin Ding,[1] Yahong Liu,[1] Chunrong Luo,[1] Xiaopeng Zhao,[1] & Jiangfeng Zhou[2]

[1]Department of Applied Physics, Northwestern Polytechnical University, Xi'an, 710129, China.

[2]Department of Physics, University of South Florida, 4202 East Fowler Ave, Tampa, FL, 33620-5700. Correspondence and requests for materials should be addressed to K.S. (email: songkun@nwpu.edu.cn), J.Z. (email: jiangfengz@usf.edu) or X.Z. (email: xpzhao@nwpu.edu.cn)

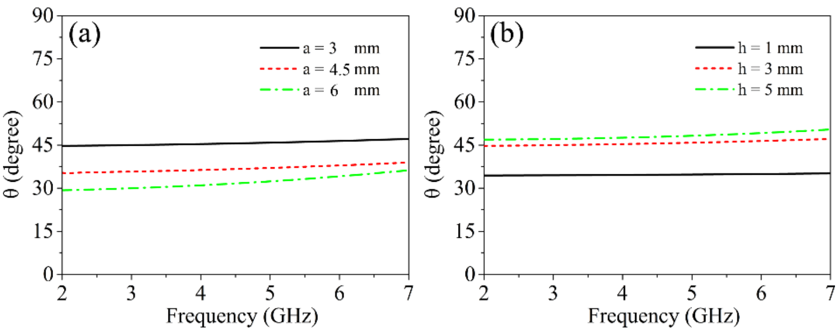

Figure S1. Dependence of azimuth rotation angle, θ, on the geometrical size a and spacing h. Here θ is calculated from simulation data.

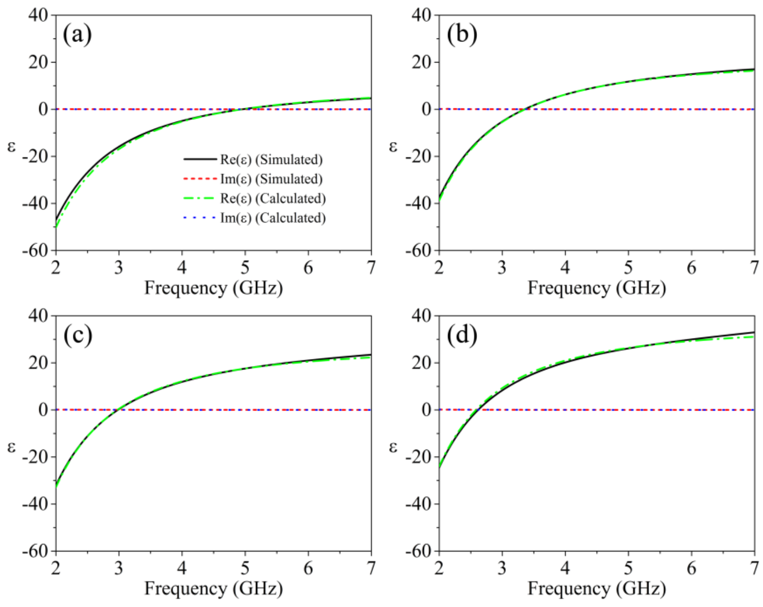

Figure S2. The simulated and calculated effective permittivity of the single-layer CMM with different dielectric substrates. (a) Air, (b) F4BM-2, (c) Taconic RF-35, (d) FR-4. The permittivity of air, F4BM-2, Taconic RF-35, and FR-4 substrates are 1.0+0.00**i*, 2.65+0.001**i*, 3.5+0.001**i*, and 4.6+0.001**i*, respectively. In the calculations, the quantitative fitting parameters for the case of air, F4BM-2, Taconic RF-35, and FR-4 substrates are chosen as $\begin{cases}\varepsilon_f = 9.82 \\ \mu_f = 0.97\end{cases}$, $\begin{cases}\varepsilon_f = 21.3 \\ \mu_f = 0.95\end{cases}$, $\begin{cases}\varepsilon_f = 27.2 \\ \mu_f = 0.92\end{cases}$, and $\begin{cases}\varepsilon_f = 35.1 \\ \mu_f = 0.90\end{cases}$, respectively.

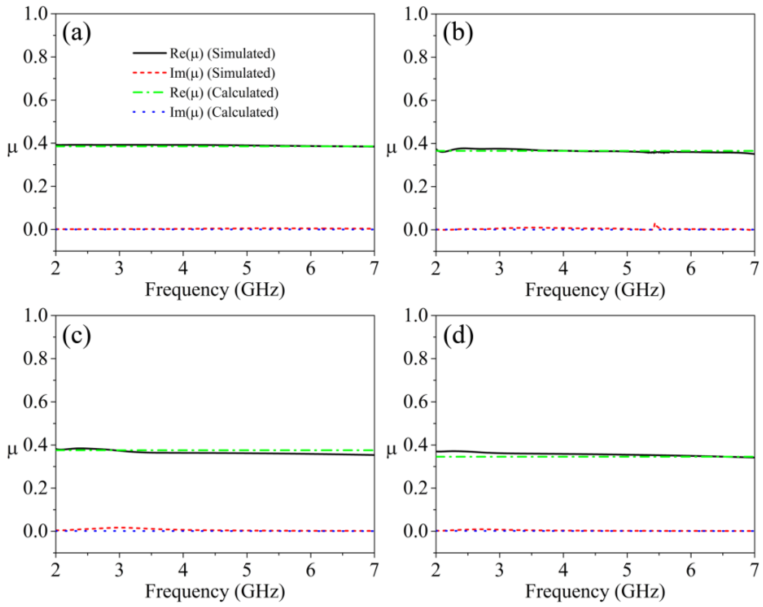

Figure S3. The simulated and calculated effective permeability of the single-layer CMM with different dielectric substrates. (a) Air, (b) F4BM-2, (c) Taconic RF-35, (d) FR-4.

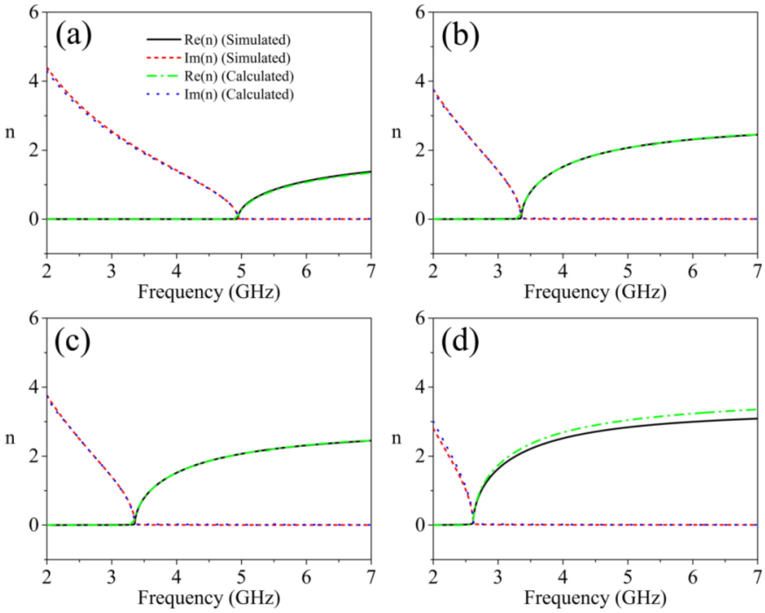

Figure S4. The simulated and calculated effective refractive index of the single-layer CMM with different dielectric substrates. (a) Air, (b) F4BM-2, (c) Taconic RF-35, (d) FR-4.

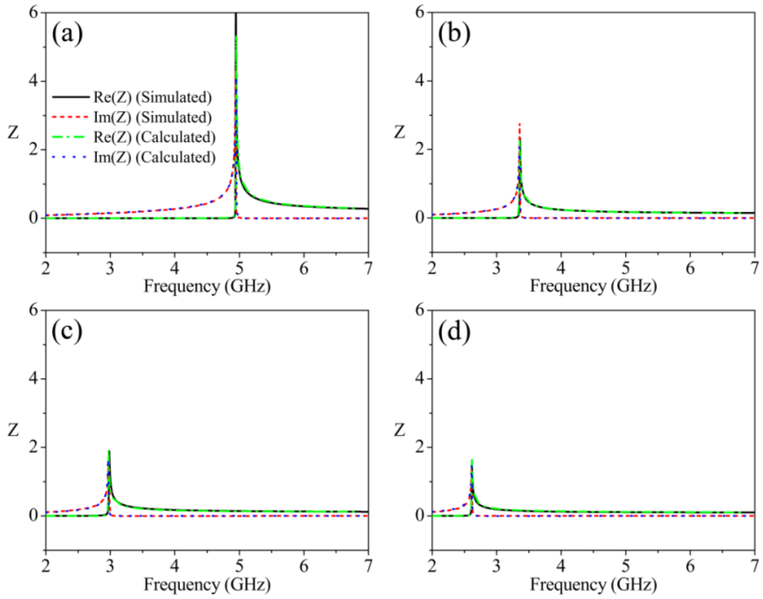

Figure S5. The simulated and calculated effective impedance of the single-layer CMM with different dielectric substrates. (a) Air, (b) F4BM-2, (c) Taconic RF-35, (d) FR-4.

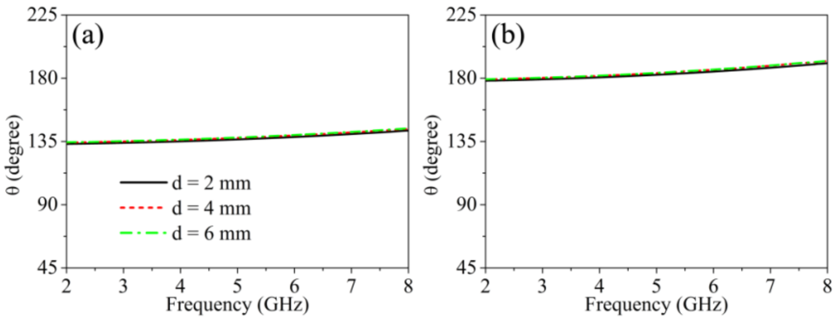

Figure S6. The simulated polarization azimuth rotation angle of the multi-layer CMMs. (a) Three layers, (b) Four layers.
